# Extracellular Nicotinamide Phosphoribosyltransferase Is a Component of the Senescence-Associated Secretory Phenotype

**DOI:** 10.3389/fendo.2022.935106

**Published:** 2022-07-14

**Authors:** Chisaka Kuehnemann, Kang-Quan Hu, Kayla Butera, Sandip K. Patel, Joanna Bons, Birgit Schilling, Cristina Aguayo-Mazzucato, Christopher D. Wiley

**Affiliations:** ^1^ Jean Mayer USDA Human Nutrition Research Center on Aging at Tufts University, Boston, MA, United States; ^2^ Buck Institute for Research on Aging, Novato, CA, United States; ^3^ Joslin Diabetes Center, Harvard Medical School, Boston, MA, United States

**Keywords:** senescence, cellular senescence, NAMPT, eNAMPT, SASP, NAD, aging, diabetes

## Abstract

Cellular senescence is a stress or damage response by which a cell adopts of state of essentially permanent proliferative arrest, coupled to the secretion of a number of biologically active molecules. This senescence-associated secretory phenotype (SASP) underlies many of the degenerative and regenerative aspects of cellular senescence - including promoting wound healing and development, but also driving diabetes and multiple age-associated diseases. We find that nicotinamide phosphoribosyltransferase (NAMPT), which catalyzes the rate-limiting step in nicotinamide adenine dinucleotide (NAD) biosynthesis, is elevated in senescent cells without a commensurate increase in NAD levels. This elevation is distinct from the acute DNA damage response, in which NAD is depleted, and recovery of NAD by NAMPT elevation is AMPK-activated protein kinase (AMPK)-dependent. Instead, we find that senescent cells release extracellular NAMPT (eNAMPT) as part of the SASP. eNAMPT has been reported to be released as a catalytically active extracellular vesicle-contained dimer that promotes NAD increases in other cells and extends lifespan, and also as free monomer that acts as a damage-associated molecular pattern and promotes conditions such as diabetes and fibrosis. Senescent cells released eNAMPT as dimer, but surprisingly eNAMPT appeared in the soluble secretome while being depleted from exosomes. Finally, diabetic mice showed elevated levels of eNAMPT, and this was lowered by treatment with the senolytic drug, ABT-263. Together, these data reveal a new SASP factor with implications for NAD metabolism.

## Introduction

Age is the greatest risk factor for the development of multiple degenerative conditions including cancer, cardiovascular disease, pulmonary fibrosis, Alzheimer’s disease, and diabetes (1, 2). The increase in incidence of these conditions with age suggests that common mechanisms that underlie most or all of aging might drive these diseases, but also implies that multiple morbidities might be treatable by targeting these common mechanisms. Two common features of aging include the accumulation of senescent cells and the loss of NAD in aged tissue (3–5). Importantly, interventions that target either of these features prevent multiple age-related conditions in animal models (6, 7).

Cellular senescence is a basic aging process that promotes multiple degenerative, age-related conditions. While a major feature of senescence is an essentially permanent arrest of cellular proliferation, senescent cells also release a myriad of biologically active molecules in the form of secreted proteins, oxylipins, exosomes, and other factors collectively known as the senescence-associated secretory phenotype (SASP). Transgenic and pharmacological interventions that selectively kill senescent cells (senolytics) prevent multiple degenerative pathologies, indicating that senescent cells and the SASP can drive these diseases (6, 8–13). For example, the BCL-2/w/xL inhibitor ABT-263 eliminates senescent cells, improving conditions such as myeloid suppression, pulmonary fibrosis, insulin resistance and diabetes (8, 14, 15). Conversely, senescent cells also have beneficial effects, including promotion of normal embryogenesis, wound healing, and parturition (16–19), so elimination of senescent cells may not be desirable in some contexts.

Levels of NAD decrease with age across multiple tissues (3, 4), due at least in part to the activity of the ectoenzyme CD38, which converts NAD to nicotinamide and cyclic-ADP ribose (20). NAD levels are maintained in most tissues *via* the NAD salvage pathway and its rate-limiting enzyme, nicotinamide phosphoribosyltransferase (NAMPT), which catalyzes the conversion of nicotinamide to nicotinamide mononucleotide (NMN). NAMPT and NMN have been shown to antagonize multiple degenerative pathologies including diabetes (21, 22), pseudohypoxia (3), and neurocognitive dysfunction (23). NAMPT is also released into the extracellular space (eNAMPT) by hepatocytes (24), macrophages (25), cancer cells (24, 26), cardiomyocytes (27) and many other cell types. Adipose tissue releases eNAMPT in the form of small extracellular vesicles (EVs) which can be endocytosed by recipient cells to elevate NAD in other tissues, preventing diabetes (22), influencing behavior and neural activity (28, 29), and extending lifespan (22). Conversely, eNAMPT also occurs in a non-vesicle contained, monomeric form that acts as a damage-associated molecular pattern (DAMP), drives macrophage activation and survival (30–32), and promotes degenerative conditions such as pulmonary fibrosis and, ironically, diabetes (33–35). Thus, much like senescent cells, eNAMPT can have beneficial or detrimental effects depending on how it is packaged.

Loss of NAD and cellular senescence are notably interconnected. For example, both decreases in NAD+/NADH ratios and depletion of NAD+ can drive senescence, but also limit parts of the SASP (36, 37). Furthermore, supplementation with the NAD precursor, nicotinamide riboside, prevents cellular senescence and extends lifespan in mice (7). Senescent cells, through their SASP, can also drive age-related NAD depletion by activating macrophages, resulting in CD38 activation (38, 39). Thus, NAD metabolism and senescence are clearly linked. However, the relationship between senescence and eNAMPT is less clear.

Here we show that senescent cells have increased levels of NAMPT. However, we do not observe a commensurate increase in NAD levels. Instead, senescent cells release eNAMPT as part of the SASP, and it is primarily released as a dimer. Furthermore, in a mouse model of diet-induced diabetes, we find that eNAMPT is increased, and is in turn lowered by the senolytic compound ABT-263. Our results highlight a new feature of senescent cells and identify a new mechanism by which senescent cells might drive or prevent degenerative pathologies.

## Materials and Methods

### Cell Culture and Induction of Senescence

IMR-90 human fibroblasts (ATCC) were cultured in Dulbecco’s Modified Eagle Medium (DMEM) supplemented with 10% fetal bovine serum (FBS) and penicillin/streptomycin. Unless otherwise stated, all non-senescent cells were quiescent for this study. Quiescence was induced by reducing FBS to 0.2% for at least 48 hours, and senescent cells were similarly treated prior to analysis. Senescence was induced by 10 Gy ionizing radiation [SEN(IR)], lentiviral shRNA-mediated depletion of sirtuin 3 [shSIRT3], lentiviral overexpression of constitutively active HRAS [RasV12] (RRID : Addgene_22262), or 10 days of continuous culture in 1 mM sodium butyrate (NaBu) or 500 nM antimycin A (MiDAS). Bleomycin-treated cultures were described previously (40). Briefly, cells were treated with either 100 ug/mL bleomycin or a matching volume of PBS stock in growth media for 3 hours at 20% oxygen. Media was then changed and cells were returned to 3% oxygen. Bleomycin-treated cells were analyzed 10 days after treatment. All cultures were considered senescent if they showed a <5% EdU labeling and > 75% senescence-associated beta-galactosidase positivity. GSE-22 (RRID : Addgene_22253) expressing cells were described previously (41). Conditioned media were generated by 24 hours of continuous culture in serum-free DMEM. All cells were confirmed mycoplasma-free.

### Quantitative Real-Time PCR

RNA was isolated from 200,000-500,000 cells using the Isolate II RNA Extraction Kit (Bioline). RNA (250 ng/μl) was used to synthesize cDNA using a High Capacity cDNA synthesis kit (Thermo) according to the manufacturer’s instructions. Gene expression was analyzed by qPCR using universal probe library primers (Roche) previously described (36) or listed below and a LighterCycler 480 II Real Time PCR System (Roche). RNA levels were normalized to beta-actin. Primer sequences were: NAMPT forward – aagggatggaactacattcttga, Reverse – ctgtgttttccaccgtgaag, UPL Probe #6; NMNAT1 forward – gaaatccctagagccaaaaaca, reverse – ggaacagcaaaggactccaa, UPL Probe #43; NMNAT2 forward – gatcctgctgctgtgtggta, reverse - cctccatatctgcctcgttc, UPL Probe #67; NMNAT3 forward – cgtttccctctcggacct, reverse – ctgctatttgcagggctca, UPL Probe #3; NAPRT1 forward – tgctctgcctggtcagcta, reverse – tctagcagccgcttctctg, UPL Probe #64; NMRK1 forward – tcctgactattccatatgaagaatgta, reverse – tggaggctgatagacccttg, Probe #63; NADK forward – acgctgctgtacgcttcc, reverse - agctgaatggggtcagga, UPL Probe #37.

### NAD Measurement

NAD was measured using a commercial kit (Biovision) according to the manufacturer’s instructions. Extractions were performed using 500,000 cells per sample homogenized in 500 μL of DNA lysis buffer, and fractionated using 10 kDa cutoff filters (Millipore) spun at 10,000 × g for 45 min. Standard curves (5–200 pg/ml) were generated for quantification.

### Western Blotting

Cells were lysed in 5% SDS in 10 mM Tris, pH 7.4, and protein content determined by BCA assay. Ten micrograms of cell lysates were loaded per well. For conditioned media, 200,000 cells were cultured in 1 mL serum-free DMEM for 24h. Media was concentrated 20-fold using 30 kDa cutoff filters spun at 10,000 x g for 20 minutes, and 100,000 cell equivalents of media were added per lane in the absence (non-reducing) or presence (reducing) of 2-mercaptoethanol. Antibodies were (Cell Signaling Technology Cat# 2531, RRID: AB_330330), (Abcam Cat# ab4074, RRID: AB_2288001) and (Abcam Cat# ab45890, No RRID). Western densitometry was quantified using ImageJ.

### eNAMPT ELISAs

For human cell culture supernatants, ~200,000 cells equivalents per mL were analyzed using a human PBEF/Visfatin ELISA (BioVision) according to the manufacturer’s instructions. For mouse sera, 50 μL of serum were diluted 1:1 with Assay Buffer and analyzed by mouse/rat eNAMPT Dual ELISA (Adipogen) according the manufacturer’s instructions.

### Quantitative proteome analysis of extracellular vesicular NAMPT

Senescence was induced in IMR90 fibroblast cells by three different stimuli: irradiation (IR;10 Gy X-ray), doxorubicin (DOXO; 250 nM, 24 hr treatment), and mitochondrial dysfunction induced senescence (MiDAS; Antimycin A; 500 nM, continuous) and separately induced quiescent as control as described previously (36, 42) SASP EV extraction was performed using size-exclusion chromatography (SEC) and ultrafiltration as detailed previously (43). Briefly, EV proteins were extracted using a SDS-based buffer, and quality check was performed by western blotting with exosome protein-specific antibodies. EV proteins were then reduced and alkylated using S-Trap mini (Protifi, Farmingdale, NY) followed by on-column digestion with trypsin (1:20 (w/w) enzyme:protein ratio). Peptides were desalted using stage tips, vacuum dried, and resuspended in aqueous 0.2% formic acid. Finally, indexed Retention Time Standards [iRT, Biognosys, Schlieren, Switzerland) (44)] were added to each sample according to the manufacturer’s instructions for mass spectrometry-based quantitative analysis.

LC-MS/MS analyses were performed on a Dionex UltiMate 3000 system coupled online to an Orbitrap Eclipse Tribrid mass spectrometer (Thermo Fisher Scientific, San Jose, CA). Samples were acquired in data-independent acquisition (DIA) mode using a 210-min chromatographic gradient. Full MS1 spectra were collected at 120,000 resolution (AGC target: 3e6 ions, maximum injection time: 60 ms, 350-1,650 m/z), and MS2 spectra at 30,000 resolution (AGC target: 3e6 ions, maximum injection time: Auto, NCE: 27, fixed first mass 200 m/z). The DIA precursor ion isolation scheme consisted of 26 variable windows covering the 350-1,650 m/z mass range with a window overlap of 1 m/z (**Supplementary Table S1**) (45). DIA data were processed in Spectronaut v15 (version 15.1.210713.50606; Biognosys) using directDIA. Data was searched against the Homo sapiens proteome with 42,789 protein entries (UniProtKB-TrEMBL), accessed on 12/07/2021. Protein identification was performed requiring a 1% q-value cutoff on the precursor ion and protein level (experiment), and 5% q-value cutoff on the protein level. Quantification was based on the peak areas of the best 3-6 MS2 fragment ions, with no normalization, and iRT profiling and q-value sparse data filtering applied. Differential protein expression analysis was performed using a paired t-test, and p-values were corrected for multiple testing, specifically applying group wise testing corrections using the Storey method (46). NAMPT (P43490) protein was identified and quantified with 4 unique peptides.

### Animals

Experiments were conducted at Joslin Diabetes Center with approval of its Animal Care and Use Committee. Mice were kept on a 12-hour light/dark cycle with water and food *ad libitum*. DIO very high fat diet (VHFD) 60kcal% fat (Fisher Scientific) was administered during 4 or 8 weeks to C57Bl6/J 8-week-old male mice acquired from Jackson Labs. Mice were treated with ABT-263 (Selleck Chemicals, in ethanol:polyethylene glycol 400:Phosal 50 PG) or vehicle. ABT-263 was administered to mice by gavage at 50 mg/kg body weight per day (mg/kg/d) for 4-5 d per cycle, with a week between the cycles during 8 weeks. For the 4 weeks HFD experiment, the period between ABT-263 cycles was increased to two weeks.

## Results

### NAMPT Levels Increase During Senescence Without Increasing Cellular NAD Levels

**Figure 1:**
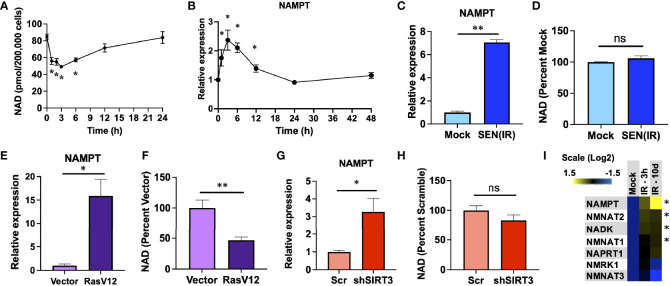
NAMPT is induced both acutely and chronically during senescence. **(A, B)**. IMR-90 fibroblasts were irradiated with 10 Gy of ionizing radiation (IR) and analyzed at the indicated timepoints for **(A)** NAD levels and **(B)** NAMPT RNA expression by quantitative PCR (qPCR). **(C, D)**. IMR-90 fibroblasts were irradiated with 10 Gy of IR [SEN(IR)] or mock-irradiated and analyzed 10 days later for **(C)** NAMPT expression, or **(D)** NAD levels. **(E, F)**. IMR-90 fibroblasts were transduced with a lentiviral RasV12 expression vector or and empty vector and analyzed for **(E)** NAMPT expression, or **(F)** NAD levels. **(G, H)**. IMR-90 fibroblasts were transduced with a lentiviral SIRT3 shRNA vector (shSIRT3) or a scrambled shRNA (Scr) and analyzed for **(G)** NAMPT expression, or **(H)** NAD levels. **(I)** RNA from **(B** and **C)** was analyzed for NAD metabolism gene expression by qPCR. All RNA measurements were normalized to beta-actin. Data are presented as means ± SEM for ≥ 3 experiments. * = p<0.05. ** = p<0.01 (t-test with Welch’s correction for **(A–H)**. One-way ANOVA for **I**). NS, non-senescent.

Genotoxic stress is a common inducer of both senescence and NAD depletion. To evaluate the relationship between genotoxic stress, NAD, and senescence - we irradiated human IMR-90 fibroblasts with 10 Gy of ionizing radiation (IR) and measured both NAD and NAMPT RNA levels over the next 24-48 hours. IR promoted an immediate acute loss of NAD over the first 3 hours, in agreement with previous studies (47, 48), followed by a gradual recovery until reaching pre-irradiation levels by 24 hours (**Figure 1A**). Since NAMPT is the rate-limiting enzyme in NAD salvage, we hypothesized that this recovery might be due to elevation of NAMPT levels. In agreement with this, *NAMPT* RNA levels increased over the first 3 hours and rapidly returned to pre-IR levels by 24 hours (**Figure 1B**). Since senescence is a chronic condition, and previous reports indicate that NAMPT is elevated during senescence (37), we also measured *NAMPT* and NAD levels in senescent cells 10 days after IR. Senescent cells had elevated *NAMPT* levels (**Figure 1C**), but this was not linked to an increase in NAD (**Figure 1D**). To determine if this was a common feature of senescent cells, we also assayed senescence induced by overexpression of constitutively active RAS (RasV12) (**Figures 1E, F**) and mitochondrial dysfunction-associated senescence (MiDAS) induced by shRNA-mediated depletion of sirtuin 3 (shSIRT3) (**Figures 1G, H**). In each case, senescence was accompanied by increased *NAMPT* levels, but no commensurate increase in NAD levels. Indeed, RAS-induced senescence was associated with lower levels of NAD (**Figure 1F**), despite strongly elevated *NAMPT* levels. We also measured RNA levels of other NAD synthetic enzymes at 3 hours and 10 days after IR, but only observed small (<2 fold) increases in these, and none showed the strong increases with senescence that we observed for NAMPT (**Figure 1I**).

### AMPK Is Required for Acute NAMPT Elevation and NAD Recovery, but Dispensable for NAMPT Expression During Senescence

**Figure 2:**
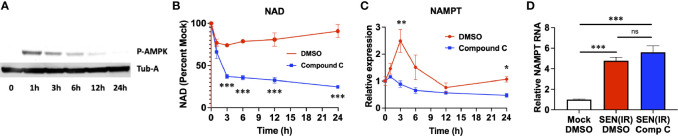
Acute, but not chronic, NAMPT induction requires AMPK activity. **(A)** IMR-90 fibroblasts were irradiated with 10 Gy of ionizing radiation (IR) and analyzed by western blot for phosphorylated AMPK and tubulin-alpha (Tub-A) at the indicated timepoints. **(B)** IMR-90 fibroblasts were irradiated with 10 Gy of ionizing radiation (IR) in the presence of DMSO or compound C and analyzed at the indicated timepoints for **(B)** NAD levels and **(C)** NAMPT expression normalized to beta-actin. **(D)** IMR-90 fibroblasts were irradiated with 10 Gy of IR [SEN(IR)] or mock-irradiated. 9 days after IR, cells were treated with either DMSO or compound C (Comp C) and analyzed for NAMPT RNA levels by quantitative PCR. All RNA measurements were normalized to beta-actin. Data are presented as means ± SEM for ≥ 3 experiments. * = p<0.05. ** = p<0.01, *** = p<0.001 (t-test with Welch’s correction) for all values. NS, non-senescent.

Loss of NAD results in AMPK activation (36, 37), and poly-ADP ribose polymerase (PARP) activity during recovery from genotoxic stress also elevates AMP levels and AMPK activation (48). We therefore sought to assess the role of AMPK in elevation of NAMPT in genotoxic stress and senescence. AMPK was rapidly phosphorylated following IR, and this phosphorylation decreased over time (**Figure 2A**). To address the role of AMPK in NAD recovery, we treated irradiated cells with an AMPK inhibitor (Compound C) (49) or vehicle (DMSO) and measured both NAD (**Figure 2B**) and *NAMPT* RNA levels (**Figure 2C**). Compound C treatment prevented recovery of NAD levels following irradiation (**Figure 2B**), and this was coupled to a failure to elevate *NAMPT* levels (**Figure 2C**). Therefore, AMPK activity is required for *NAMPT* elevation following genotoxic insult. Conversely, 24 hours of compound C treatment had no effect on *NAMPT* levels once senescence was fully established 10 days after irradiation (**Figure 2D**). Thus, distinct mechanisms elevate NAMPT during the acute DNA damage response and during chronic senescence.

### Extracellular NAMPT Is a SASP Factor

**Figure 3:**
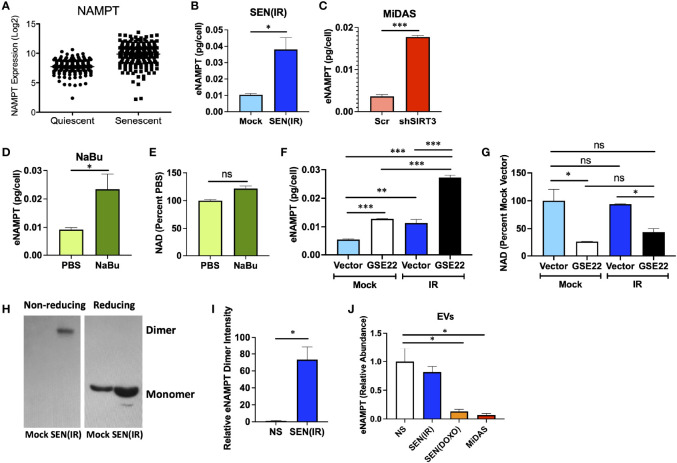
eNAMPT is a SASP factor. **(A)** PBS control non-senescent and bleomycin-induced senescent cells were analyzed for *NAMPT* RNA expression at the single cell level. [Dataset originally described in (40)]. **(B–D)** Cells were induced to senesce by IR, shSIRT3, or sodium butyrate (NaBu). Ten days after induction, cells were cultured in serum-free media for 24 h. Conditioned media was then analyzed by ELISA and normalized to cell number. **(E)** NAD levels were measured in cells from **(D)**. **(F, G)** Cells were transduced with either a GSE22 expression lentivirus or an empty vector and either mock irradiated or induced to senesce with 10 Gy of IR. Conditioned media were generated as in **(B)**, and cells were collected, counted and analyzed for **(F)**. eNAMPT secretion by ELISA, and **(G)**. NAD levels. **(H)** Conditioned media from non-senescent and senescent [SEN(IR)] cells were analyzed by western blot under non-reducing (left) and reducing (right) conditions. **(I)** Relative intensities of eNAMPT dimer blots were quantified by densitometry. **(J)** Extracellular vesicles were isolated from quiescent (QUI) or senescent cells induced by IR, doxorubicin (DOXO) or mitochondrial dysfunction (MiDAS – antimycin A) and analyzed mass spectrometry for eNAMPT. Data are presented as means ± SEM for ≥ 3 experiments. * = p<0.05. ** = p<0.01 (t-test with Welch’s correction for **A–E, I, J**. One-way ANOVA for **F, G**). NS, non-senescent.

Since NAMPT was elevated during senescence, but NAD levels were not, we considered the possibility that NAMPT is secreted by senescent cells. We first analyzed our previously reported single cell gene expression dataset (40) for *NAMPT* expression (**Figure 3A**). Unlike cyclin-dependent kinase inhibitors and housekeeping genes, SASP factors show increased variance with senescence – with only a subset of cells showing clear elevation (40). *NAMPT* expression was similar to that observed with SASP factors such as *IL8* or *IL1A* - suggesting that it might be secreted. We therefore measured extracellular NAMPT (eNAMPT) levels in conditioned media from senescent cells by ELISA. Cells induced to senesce by IR (**Figure 3B**), mitochondrial dysfunction (**Figure 3C**), and HDAC inhibition (sodium butyrate – NaBu) (**Figure 3D**) all released eNAMPT. In agreement with other inducers of senescence, NaBu–induced senescent cells also did not have elevated NAD levels (**Figure 3E**).

Release of many SASP factors is restrained by the activity of p53 (41, 50), though DAMPs such as HMGB1 are released in a p53-dependent manner during senescence (51). We therefore used a dominant negative p53 genetic suppressor element (GSE22) (52) to eliminate p53 activity and assess its role in eNAMPT release. Like many proinflammatory SASP factors, such as IL-6, elimination of p53 activity elevated eNAMPT release (**Figure 3F**). Conversely, loss of p53 activity lowered NAD levels, regardless of senescence inducer status (**Figure 3G**). Thus, eNAMPT release is regulated in a manner similar to proinflammatory SASP factors such as IL-6 or IL-1B.

eNAMPT is released in multiple forms. For example, eNAMPT is released as a catalytically active dimer in extracellular vesicles (EVs) from tissues such as visceral fat (22). This form of eNAMPT can be endocytosed by recipient cells, where NAMPT then elevates NAD levels (22). This has been shown to antagonize age-associated NAD loss, prevent diabetes, and extend lifespan in mice (22). Alternatively, eNAMPT has been reported to occur outside of EVs, where it can bind TLR4 and act as a DAMP to drive inflammation (53). The DAMP form of eNAMPT is also at least partly monomeric, as opposed to the catalytically active version which appears as a disulfide-linked NAMPT dimer (34, 35). We therefore analyzed conditioned media from non-senescent or senescent cells by western blot using reducing and non-reducing conditions, as described previously (34). Virtually all eNAMPT detected in non-reducing conditions was dimerized (**Figure 3H**), while reducing conditions showed only monomer, as expected. Under non-reducing conditions, virtually all eNAMPT was detected in conditioned media from senescent cells, and virtually none from non-senescent cells (**Figures 3H, I**).

We previously used mass spectrometry to identify new SASP factors as part of a large proteomic SASP Atlas (54). In these datasets, which also used IMR-90 fibroblasts, eNAMPT was found exclusively in the soluble secretome of conditioned media from senescent cells induced by IR or RAS (54), and no eNAMPT was found in exosomes. Since we expected at least some eNAMPT in EVs, we analyzed an additional proteomic dataset from EVs from either non-senescent cells or cells induced to senesce by IR, doxorubicin (DOXO), or mitochondrial dysfunction (MiDAS). EVs from senescent cells induced by DOXO or MiDAS showed depleted levels of eNAMPT relative to quiescent cells, although IR-induced senescence did not show significant changes (**Figure 3J**). Regardless of inducer, no EVs from senescent cells showed increases in eNAMPT, even though total eNAMPT is increased with senescence. These data indicate that eNAMPT is primarily released from senescent cells as a soluble dimer.

### Senescent Cells Release eNAMPT in Diabetic Mice

**Figure 4:**
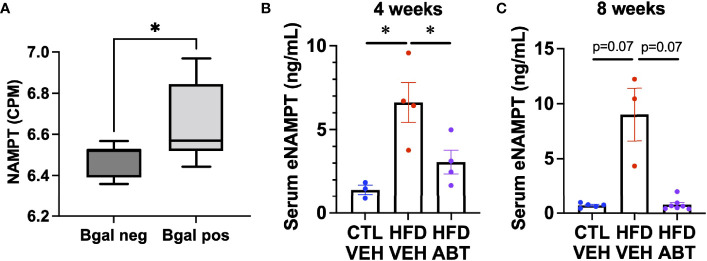
Senescent cells promote eNAMPT increases during diabetes. **(A)** Pancreases from 7-month-old mice were stained for senescence-associated beta-galactosidase, FACs sorted by beta-galactosidase positivity, and analyzed by RNA-seq for *NAMPT*, and expressed as counts per million (CPM). [Dataset previously described in (8)]. n = 7 mice. **(B, C)** Mice were fed either a normal or high fat diet and treated with either ABT-263 or vehicle *via* gavage for either **(B).** 4 weeks, or **(C).** 8 weeks. Sera from treated mice were analyzed for eNAMPT by ELISA. N’s for **(B, C)** are shown by individual data points. * = p<0.05 (t-test with Welch’s correction) for all experiments.

Diabetes is causally linked to the release of eNAMPT. Notably, EV-contained eNAMPT antagonizes diabetic phenotypes, whereas the soluble monomeric form has been shown to promote diabetes (21, 34, 35). Since senescent cells can promote diabetes, we hypothesized that they might be a source of eNAMPT during metabolic stress. To determine if pancreatic beta cells also elevate NAMPT during senescence *in vivo*, we analyzed our previously generated RNA-seq data from either beta-galactosidase positive or beta-galactosidase negative pancreatic beta cells from 7-8 month old mice for NAMPT expression (**Figure 4A**) **(** 8). We then analyzed sera from diabetic mice that were given a diabetes-inducing high fat diet (HFD) treated with either the senolytic drug ABT-263 (ABT) (14) or a vehicle for 4 weeks (**Figure 4B**) or 8 weeks by gavage (**Figure 4C**) for eNAMPT by ELISA. Animals from the 8-week study were previously described (8). In either treatment protocol, HFD elevated eNAMPT levels, and these were lowered by ABT-263 treatment. Together, these data indicate that senescent cells are a source of eNAMPT during diabetes.

## Discussion

Our results reveal a new aspect of senescent cells and a potential additional role for senescence in NAD metabolism. While it is known that senescent cells elevate NAMPT, the prevailing understanding is that this allows senescent cells to increase intracellular NAD+/NADH ratios and potentiate a highly inflammatory state by antagonizing AMPK activation (37). By comparison, the SASP activates macrophages and increases CD38 levels and activity, and thereby drives age-related NAD depletion. Here we show that NAMPT itself is a SASP factor. Both the SASP and eNAMPT promote some of the same diseases, including diabetes (8, 10, 12, 34, 35) and pulmonary fibrosis (9, 11, 15, 33), so it is appealing to consider senescent cells as a source of eNAMPT in these diseases.

Since eNAMPT is reported to exist in at least 2 states – EV-contained or a free soluble form – the effects of eNAMPT can either be anti-diabetic and anti-geronic by elevating NAD levels *via* the EV-contained form (22), or diabetogenic and disease-driving *via* activation of TLR4 in a soluble and/or monomeric form (33–35, 53). Further, eNAMPT can also promote M1 polarization of macrophages in a TLR4-independent manner (32). Ironically, since M1 polarization of macrophages elevates CD38 levels (38), it is possible that free eNAMPT monomers may actually *lower* NAD levels *in vivo*, and the secretions of senescent cells are known to do this (38). Importantly, previous work showed that even though the predominant form of eNAMPT in biological fluids is dimeric, the shift from dimer to monomer can drive diabetes, and soluble eNAMPT undergoes a dose-dependent conformation shift that promotes this process (34, 35). Since it is still unclear if the eNAMPT released by senescent cells retains its catalytic activity, the precise nature and effects of senescence-derived eNAMPT are a potentially important area for future study.

Our results also imply a potentially novel mechanism that could be targeted for aging, diabetes, and related conditions. If the eNAMPT produced by senescent cells could be shifted into its EV-contained form, senescent cells might be able to elevate NAD levels in surrounding tissues. Additionally, if this prevents free eNAMPT from binding and activating TLR4 in macrophages, this might lower CD38 levels and prevent age-related NAD depletion. In this way, identification of the mechanism that determines whether eNAMPT appears as a free versus a vesicle-contained protein could potentially be exploited to shift cellular senescence from a NAD-depleting process to a NAD-increasing process. Notably, blocking antibodies to eNAMPT have been used to prevent multiple degenerative diseases including pulmonary fibrosis (55), diabetes (34), and colitis (56). Such interventions would hold strong therapeutic potential for the treatment of diabetes and other senescence-driven degenerative diseases.

## Data Availability Statement

The datasets presented in this study can be found in online repositories. The names of the repository/repositories and accession number(s) can be found below: https://www.ncbi.nlm.nih.gov/, GSE121539 http://www.proteomexchange.org/, PXD023897.

## Ethics Statement

The animal study was reviewed and approved by Joslin Diabetes Center Animal Care and Use Committee.

## Author Contributions

CW conceived of the concept, designed the experiments, interpreted the data, and wrote the manuscript. CK provided conceptual analyses and coordinated multi-site collaborations. K-QH and KB performed experiments. SP and JB conducted mass spectrometry-based proteomic on extracellular vesicles from senescent cells and analysed the data under the guidance of BS. CA-M conducted the diabetic animal experiments. All authors contributed to the article and approved the submitted version.

## Funding

This work was supported by NIH grants P01 AG017242 (PI: Judith Campisi), U01 AG060906 (PI: Schilling) and USDA-ARS cooperative agreement 58-8050-9-004. We also acknowledge the support of instrumentation for the Orbitrap Eclipse Tribrid from the NCRR shared instrumentation grant 1S10 OD028654 (PI: Birgit Schilling).

## Conflict of Interest

The authors declare that the research was conducted in the absence of any commercial or financial relationships that could be construed as a potential conflict of interest.

## Publisher’s Note

All claims expressed in this article are solely those of the authors and do not necessarily represent those of their affiliated organizations, or those of the publisher, the editors and the reviewers. Any product that may be evaluated in this article, or claim that may be made by its manufacturer, is not guaranteed or endorsed by the publisher.
